# Curcumin Ameliorates Diabetic Nephropathy by Suppressing NLRP3 Inflammasome Signaling

**DOI:** 10.1155/2017/1516985

**Published:** 2017-01-17

**Authors:** Miaomiao Lu, Nanchang Yin, Wei Liu, Xiangfei Cui, Shuo Chen, Ermin Wang

**Affiliations:** ^1^Department of Nephrology, The First Affiliated Hospital of Jinzhou Medical University, Jinzhou 121000, China; ^2^Department of Chest Surgery, The First Affiliated Hospital of Jinzhou Medical University, Jinzhou 121000, China; ^3^Department of Oncology, The First Affiliated Hospital of Jinzhou Medical University, Jinzhou 121000, China

## Abstract

Diabetic nephropathy (DN) is the leading cause of end-stage renal disease, partly because of the lack of effective treatments for DN. Curcumin has been shown to exert strong antifibrotic effects in DN, but the underlying mechanisms are not well characterized. In this study, we sought to determine the effects of curcumin on diabetic renal disease in db/db mice and characterize the underlying mechanism of action. We administered curcumin to db/db mice for 16 weeks. In comparison to mock-treated db/db mice, curcumin-treated mice showed diminished renal hypertrophy, reduced mesangial matrix expansion, and a lower level of albuminuria. Furthermore, the upregulated protein and mRNA expressions of collagen IV and fibronectin in the renal cortices of the db/db mice were inhibited by curcumin treatment. Additionally, curcumin treatment was associated with significant reductions in mature interleukin-1*β*, cleaved caspase-1, and NLRP3 protein levels in the renal cortices of db/db mice as well as in HK-2 cells exposed to high glucose concentration. In summary, curcumin, a potent antifibrotic agent, is a promising treatment for DN, and its renoprotective effects appear to be mediated by the inhibition of NLRP3 inflammasome activity.

## 1. Introduction

The incidence of diabetes is rapidly increasing worldwide, and the condition is projected to affect 300 million people by 2025, approximately one-third of whom will develop diabetic nephropathy (DN) [1]. DN is manifested by the accumulation of extracellular matrix proteins and an irreversible decline in renal function and is the leading cause of end-stage renal disease (ESRD) [2]. The molecular mechanisms underlying DN are incompletely characterized, and, thus, few clinical therapies are available for this condition [3]. Currently, the main treatment methods for DN focus on lowering blood glucose levels and reducing hypertension by inhibiting the renin-angiotensin system [4–6]. However, these approaches merely delay DN progression and therefore the development of ESRD but do not prevent ESRD [7, 8]. Thus, novel therapies that target additional DN pathways are urgently required.

Curcumin, a major component extracted from the rhizome* Curcuma longa*, commonly known as turmeric, has been consumed by humans as a curry spice for centuries. Its chemopreventive effects have been extensively investigated and are well defined [9]. A recent experimental study showed that short-term curcumin treatment in high-fat diet-fed mice ameliorated muscular oxidative stress by activating Nrf2 and disrupting the Nrf2-Keap1 complex and led to increases in the expression and activity of heme oxygenase-1 in porcine renal epithelial proximal tubule cells [10, 11]. Moreover, curcumin treatment has been shown to decrease macrophage infiltration in the kidneys of chronic renal failure rats, indicating that the anti-inflammatory properties of curcumin may be responsible for alleviating disease in this animal model [12]. Because of its strong anti-inflammatory activity, curcumin has been used to treat various inflammatory diseases such as DN, Alzheimer disease, and major depression [13]. However, the precise mechanisms by which curcumin ameliorates DN remain unclear.

Tubulointerstitial inflammation is crucial in promoting the development and progression of DN [14]. The NOD-like receptor 3 (NLRP3) inflammasome is a molecular platform activated upon signs of cellular “danger” [15]. This inflammasome is composed of the NLRP3 protein, caspase-1, and the adaptor protein apoptosis-associated speck-like protein containing a caspase-activating recruitment domain. Upon activation, the NLRP3 inflammasome triggers innate immune defenses through the maturation of proinflammatory cytokines such as interleukin-1*β* (IL-1*β*) and might contribute to the development of DN [15]. This inflammasome is activated in the kidneys of streptozotocin-induced diabetic rats, and the suppression of its activation can significantly reduce renal tissue inflammation and improve renal function in these rats [16]. Furthermore, NLRP3 knockout protects unilateral ureteral occlusion mice and renal ischemia/reperfusion-induced acute kidney injury mice from renal tubular damage and interstitial inflammation [17, 18]. In vitro experiments have shown that NLRP3 protein expression, cleavage of caspase-1 and IL-1*β*, and release of IL-1*β*, IL-18, and ATP in HK-2 cells significantly increased after high glucose stimulation [14]. These data show that the NLRP3 inflammasome plays a key role in the process of sterile kidney inflammation, which led us to hypothesize that IL-1*β* and the NLRP3 inflammasome may be responsible for the renoprotective effects of curcumin in DN.

In this study, we aimed to determine the effects of curcumin on diabetic kidney disease in db/db mice, which develop renal lesions similar to those seen in patients with DN [19]. Furthermore, we aimed to characterize the mechanisms underlying the action of curcumin by evaluating the changes in IL-1*β* expression levels and NLRP3 inflammasome activity in cultured HK-2 cells exposed to high glucose concentrations and treated with curcumin.

## 2. Materials and Methods

### 2.1. Reagents

Curcumin was purchased from Sigma-Aldrich (St. Louis, MO, USA). Antibodies were obtained from the following sources: NLRP3 antibody, Adipogen (San Diego, CA, USA); IL-1*β* and caspase-1 antibodies, Santa Cruz Biotechnology (Santa Cruz, CA, USA); collagen IV and fibronectin antibodies, Abcam (Cambridge, MA, USA); and secondary antibodies and *β*-actin antibody, Sigma-Aldrich (St. Louis, MO, USA). Enzyme-linked immunosorbent assay (ELISA) kits for mouse albumin were acquired from Assaypro (St. Charles, MO, USA). Amicon® Ultra-4 Centrifugal Filter Devices were purchased from Millipore (Billerica, MA, USA). All cell culture reagents were obtained from Invitrogen (Carlsbad, CA, USA).

### 2.2. Animals and Experimental Protocol

Male C57BL/KsJ db/db (diabetic) mice and age-matched db/m (nondiabetic) mice were purchased from the Model Animal Research Center of Nanjing University (Nanjing, China). The mice were divided into three groups of 6 mice each: a control group of db/m mice and two groups of db/db mice subjected to a mock treatment or curcumin administration. Treatments were scheduled to begin at the age of 10 weeks and end at the age of 26 weeks. All treatments were performed daily by oral gavage. Nondiabetic (db/m) and mock-treated diabetic (db/db) mice received 1% sodium carboxymethyl cellulose (a vehicle), while the remaining db/db mice received 200 mg/kg/day curcumin.

The animals were housed in a pathogen-free environment with a 12-hour light-dark cycle and given unrestricted access to standard mouse chow and water ad libitum (Department of Laboratory Animals, Central South University, Changsha, China). We measured blood glucose levels in all experimental animals once a week during the experimental period. Serum samples were collected prior to animal sacrifice at 26 weeks of age. In addition, 24-hour urine samples were collected, and urinary albumin excretion was determined (micrograms per 24 hours) as reported previously [20, 21]. All mice were sacrificed by cervical dislocation while under gaseous anesthesia (isoflurane) at 26 weeks of age, and their kidneys were harvested. The protocols for animal experimentation and the care of animals were consistent with the licenses held by the Central South University, which fulfill and follow international rules and guidelines.

### 2.3. Measurement of Urine Albumin

Mice were individually housed in metabolic cages during the last 3 days of treatment, and after 3 h of habituation, 24-hour urine samples were collected. Animals continued to have free access to water and standard laboratory diet during this period. The 24-hour urine samples were stored at −70°C until analysis. Urinary albumin concentration was measured using a mouse albumin ELISA kit (Assaypro, USA) following the manufacturer's protocol.

### 2.4. Analysis of Blood Glucose and Renal Function

After a 12-hour fast, the mice were anesthetized with chloral hydrate, and blood was obtained from the tail vein. Blood glucose was measured in duplicate using a glucose meter (OneTouch Ultra, LifeScan, Milpitas, CA, USA). The serum creatinine (SCR) level and blood urea nitrogen (BUN) level were determined with an automatic analyzer (model 7170; Hitachi Co., Ltd., Japan).

### 2.5. Histological Evaluation

After the animals were sacrificed, their kidneys were rapidly dissected and weighed, and the renal cortices were separated. The left cortices were snap-frozen for Western blot and real-time polymerase chain reaction (PCR) analyses, and the right cortices were used to histologically evaluate renal lesions. The right cortices were fixed in 10% paraformaldehyde and embedded in paraffin, and 3 *μ*m thick sections were prepared. The sections were stained with Periodic Acid-Schiff (PAS). All histological evaluations were performed in a blinded manner. Twenty glomeruli were evaluated for each mouse. The degree of damage in each glomerulus was assessed using a semiquantitative scoring method as follows: grade 0, normal glomeruli (no damage); grade 1, mesangial expansion area, up to 25% (minimal damage); grade 2, 26%–50% expansion (moderate damage); grade 3, 51%–75% expansion (moderate-to-severe damage); and grade 4, 76%–100% expansion (severe damage). The glomerular matrix expansion index (GMI) was then calculated by the following formula:

(1) $$\begin{array}{c} GMI=\frac{\left(1\times n_{1}+2\times n_{2}+3\times n_{3}+4\times n_{4}\right)}{\left(n_{0}+n_{1}+n_{2}+n_{3}+n_{4}\right)}, \end{array}$$

where *n* represents the number of glomeruli with the respective grades of damage [21].

### 2.6. Immunohistochemistry

Immunohistochemical analyses were conducted to determine the collagen IV and fibronectin levels in paraffin-embedded renal tissue sections. Pepsin-based antigen retrieval was carried out. Given the homogeneity of the target protein staining, the interstitial staining for collagen IV and fibronectin was measured using computerized morphometry (Image Pro-Plus 6.0 software, Bethesda, MD). The areas of collagen IV and fibronectin staining in 20 randomly selected fields at 400x magnification in the cortex and outer medulla were quantified as the percentage of the total measured area. The immunohistochemical assessments were performed by an observer who was blinded to the study groups.

### 2.7. Cell Culture and Stimulation

HK-2 cells were purchased from American Type Culture Collection (Rockville, MD, USA). The cells were cultured in low-glucose Dulbecco's Modified Eagle's Medium supplemented with 5% fetal bovine serum, 100 *μ*g/mL streptomycin, and 100 U/mL penicillin (Life Technologies) at 37°C in a humidified atmosphere containing 5% CO_2_. In order to find the most appropriate culture conditions, HK-2 cells were grown in media with normal glucose concentration (5 mM), high glucose concentrations (15, 25, 35, and 50 mM), or high mannitol concentrations (5 mM glucose + 30 mM mannitol) for 12, 24, 48, and 72 h with and without curcumin (5, 10, and 15 *μ*M). On the basis of the results obtained, HK-2 cells were incubated in media with 35 mM glucose with or without 10 *μ*M curcumin for 48 h in subsequent experiments to detect fibronectin, IL-1*β*, caspase-1, and NLRP3 protein expression. Cell extracts and precipitated supernatants were analyzed using ELISA and Western blot. Each experiment was repeated three times, and the average of the three values was used.

### 2.8. Western Blot Analysis

Proteins from renal cortical tissues, cell-free supernatants (extracted using the Amicon Ultra-4 Centrifugal Filter Device), and cell lysates were separated using 10% or 15% sodium dodecyl sulfate polyacrylamide gel electrophoresis and transferred to polyvinylidinedifluoride membranes (Millipore, Bedford, MA, USA). Nonspecific binding was blocked by incubation with 5% skim milk for 1 h at room temperature. The membranes were incubated overnight at 4°C with primary antibodies against fibronectin (1 : 400), IL-1*β* (1 : 400), caspase-1 (1 : 200), and NLRP3 (1 : 1000) and subsequently hybridized with horseradish peroxidase-conjugated secondary antibodies for 1 h at room temperature. The protein bands were visualized with an enhanced chemiluminescence kit and quantified using ImageJ software.

### 2.9. Quantitative Real-Time PCR

Total RNA was isolated from kidney tissues by using TRIzol reagent according to the manufacturer's instructions (Invitrogen). In total, 1 *μ*g RNA was converted to single-stranded cDNA by using SuperScript Reverse Transcriptase II (Fermentas). The resulting cDNA was amplified using the PCR SuperMix kit (TaKaRa). The following primers were used: collagen IV, 5′-AGAAGCGAGATGTTCAAGAAG-3′ (forward) and 5′-GTTGTGACGGTGGCAGAG-3′ (reverse); fibronectin, 5′-TGTTATGGAGGAAGCCGAGGTT-3′ (forward) and 5′-CGATGCAGGTACAGTCCCAGA-3′ (reverse); and *β*-actin, 5′-TGACGTGGACATCCGCAAAG-3′ (forward) and 5′-CTGGAAGGTGGACAGCGAGG-3′ (reverse). Amplification reactions were performed as follows: one cycle of 94°C for 2 min, 30 cycles of 94°C for 30 s, 60°C for 30 s, and 72°C for 1 min, followed by one cycle of 72°C for 7 min. All reactions were performed in the CFX™ Real-Time System (Bio-Rad Laboratories, Inc.). The relative abundance of mRNA was standardized with *β*-actin mRNA as the invariant control.

### 2.10. Statistical Analyses

All data were expressed as means ± standard deviation. Statistical analysis was performed using SPSS 16.0 software (SPSS Inc., Chicago, IL, USA). Comparisons among different groups were made using one-way analysis of variance. *p* < 0.05 was considered statistically significant.

## 3. Results

### 3.1. Effect of Curcumin on Renal Hypertrophy

Body and kidney weights were markedly greater in the mock-treated db/db (diabetic) mice than in the db/m (nondiabetic) control mice. The kidney : body weight ratio, however, was slightly but not significantly lower in the mock-treated db/db mice than in the db/m mice because the diabetic mice were much heavier than the nondiabetic mice. Body and kidney weights were lower in the curcumin-treated db/db mice than in the mock-treated db/db mice.

### 3.2. Effects of Curcumin on Blood Glucose Level

All db/db mice remained hyperglycemic throughout the experimental period (data not shown). Blood glucose levels were remarkably higher in the db/db mice than in the db/m mice but did not differ between the curcumin-treated and mock-treated db/db mice (Table 1).

### 3.3. Effects of Curcumin on Renal Function

The db/db mice exhibited macroalbuminuria. The urinary albumin excretion rate was 18-fold higher in the mock-treated db/db mice than in the db/m mice. The administration of curcumin was associated with a significant attenuation of albuminuria as compared to the level in the mock-treated db/db mice. In addition, the SCR level, a marker of glomerular filtration, was significantly lower in the curcumin group than in the mock treatment group. The BUN level was slightly, but not significantly, lower in the curcumin group than in the mock treatment group (Table 1).

### 3.4. Effects of Curcumin on Renal Histology

**Figure 1:**
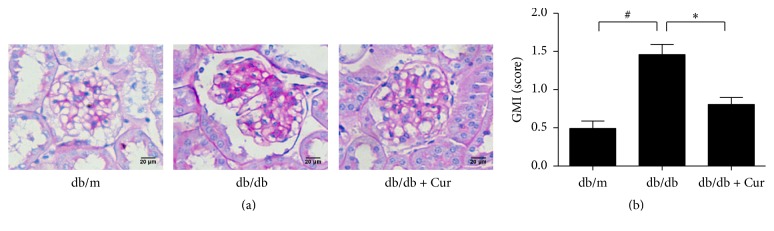
Curcumin offers protection against glomerular expansion in diabetic nephropathy. (a) Representative photomicrographs of Periodic Acid-Schiff- (PAS-) stained renal cortical sections from db/m mice, mock-treated db/db mice, and curcumin-treated db/db mice. (b) Glomerular matrix expansion index (GMI) scores calculated from the analysis of 20 glomeruli per mouse. db/m, nondiabetic mice; db/db, diabetic mice (mock-treated); db/db + Cur, db/db mice treated with curcumin at a dose of 200 mg/kg body weight. ^#^*p* < 0.05, compared with the db/m mice; ^*∗*^*p* < 0.05, compared with the mock-treated db/db mice (*n* = 6 per group). Magnification, ×400; scale bar, 20 *μ*m for all micrographs.

Mesangial matrix expansion was evident in the glomeruli of the mock-treated db/db mice (Figure 1). PAS-positive mesangial matrix areas were substantially larger in the mock-treated db/db mice than in the db/m mice. The GMI score was significantly lower in the curcumin-treated db/db mice than in the mock-treated db/db mice.

### 3.5. Effects of Curcumin on Collagen IV and Fibronectin Expression

**Figure 2:**
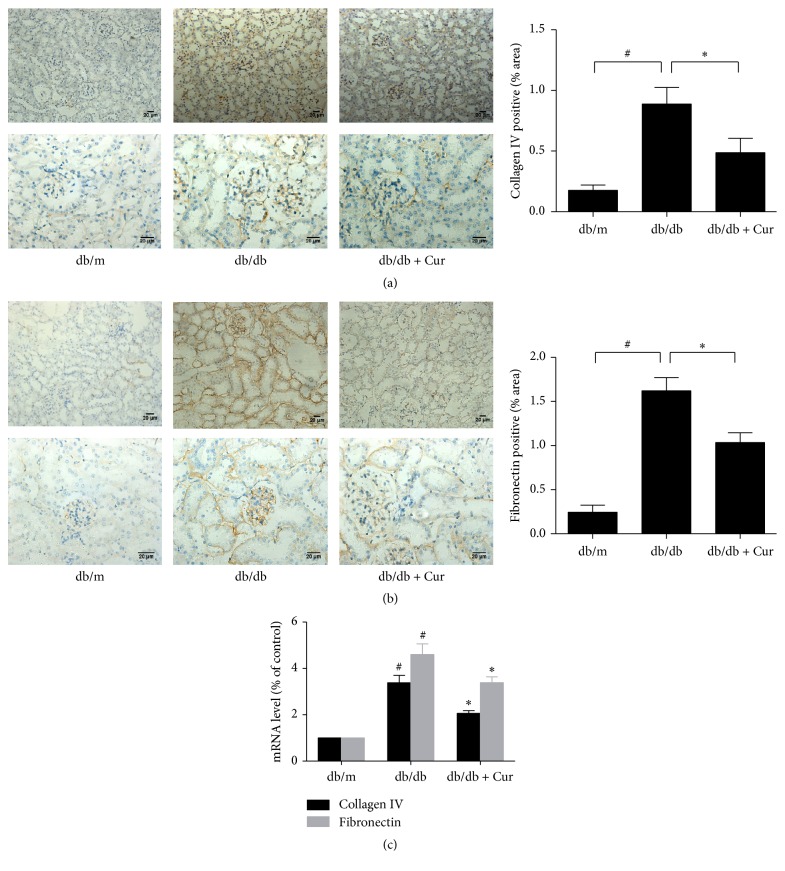
Curcumin partially reverses the upregulation of collagen IV and fibronectin expression in db/db mice. (a) Collagen IV and (b) fibronectin protein expression in the renal cortex as determined using immunohistochemical staining. Magnification, ×200 (top) and ×400 (bottom); scale bar, 20 *μ*m. (c) The mRNA levels of collagen IV and fibronectin measured using real-time polymerase chain reaction and expressed as a percentage of the mRNA level in the db/m group. db/m, nondiabetic mice; db/db, diabetic mice (mock-treated); db/db + Cur, db/db mice treated with curcumin at a dose of 200 mg/kg body weight. ^#^*p* < 0.05, compared with the db/m mice (control); ^*∗*^*p* < 0.05, compared with the mock-treated db/db mice (*n* = 6 per group).

Collagen IV and fibronectin accumulation was observed in the mesangial area of the glomeruli in mock-treated db/db mice, and both proteins were expressed at lower levels in the glomeruli of curcumin-treated db/db mice than in the mock-treated mice. The semiquantitative scores for collagen IV and fibronectin were higher in the mock-treated db/db mice than in the db/m mice and significantly lower in the curcumin-treated db/db mice than in the mock-treated db/db mice (Figures 2(a) and 2(b)). Consistent with this, the mRNA levels of collagen IV and fibronectin in the renal cortex were higher in the mock-treated db/db mice than in the db/m mice, and curcumin administration was associated with a significant reduction in these levels as determined by real-time PCR (Figure 2(c)).

### 3.6. Effects of Curcumin on IL-1*β* Production and NLRP3 Inflammasome Activity

**Figure 3:**
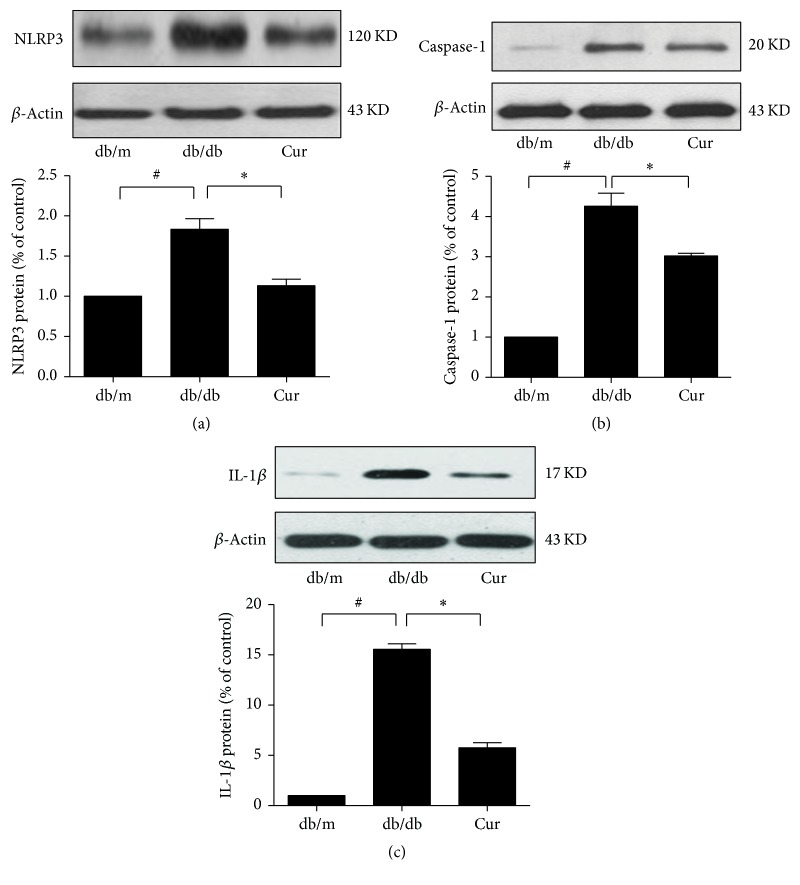
Curcumin reduced interleukin-1*β* (IL-1*β*) production and NLRP3 inflammasome activity in db/db mice. The expression of NLRP3 protein (a) and cleavage of caspase-1 (b) and IL-1*β* (c) as determined using Western blot analysis; *β*-actin was used as the internal loading control. db/m, nondiabetic mice; db/db, diabetic mice (mock-treated); db/db + Cur, db/db mice treated with curcumin at a dose of 200 mg/kg body weight. ^#^*p* < 0.05, compared with the db/m mice (control); ^*∗*^*p* < 0.05, compared with the mock-treated db/db mice (*n* = 6 per group).

We found a significant increase in NLRP3 protein expression and cleavage of caspase-1 and IL-1*β* in the kidney tissues of mock-treated db/db mice as compared to db/m mice, and this upregulation was dramatically inhibited in the curcumin group (Figure 3).

### 3.7. Effects of High Glucose Levels on NLRP3 Inflammasome Activation in HK-2 Cells

**Figure 4:**
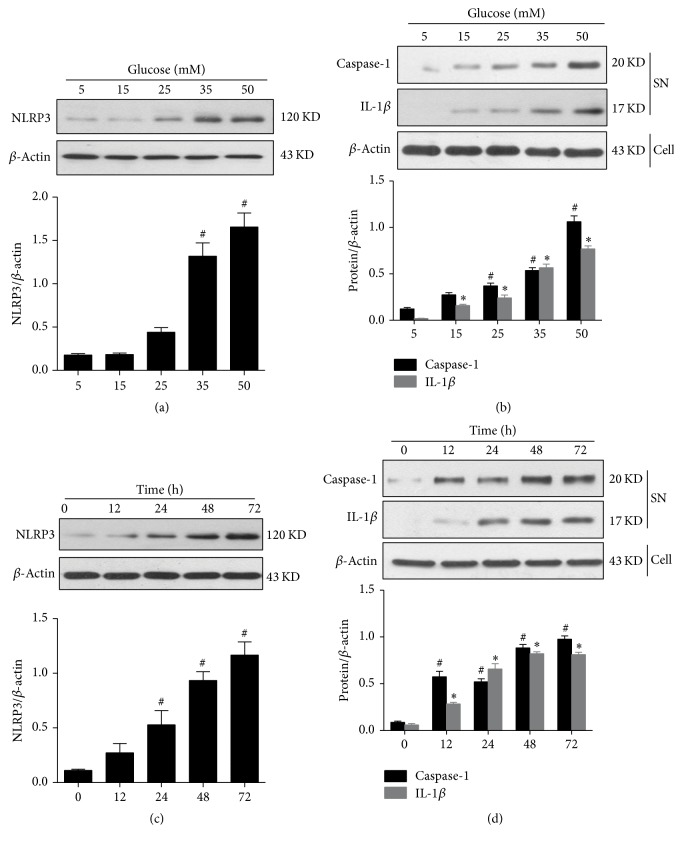
Time- and dose-dependent effects of glucose on NLRP3 inflammasome activation in HK-2 cells, as assessed using Western blot analysis. (a) NLRP3 protein expression in HK-2 cells exposed to different glucose concentrations for 48 h. (b) Cleaved caspase-1 p20 and 17 kD mature interleukin-1*β* (IL-1*β*) in culture supernatants. (c) NLRP3 protein expression in HK-2 cells treated with 35 mM glucose for different time periods. (d) Cleaved caspase-1 p20 and 17 kD mature IL-1*β* in cell culture supernatants. *β*-Actin was used as the internal loading control. (a, c) ^#^*p* < 0.05, compared with cells treated with 5 Mm glucose or cells treated for 0 h. (b, d) ^#^*p* < 0.05 and ^*∗*^*p* < 0.05, compared with cells treated with 5 mM glucose or cells treated for 0 h. The average values from three independent experiments are shown.

To investigate the effect of high glucose levels on NLRP3 inflammasome activation, we analyzed NLRP3 protein expression and the cleavage of caspase-1 and IL-1*β* in HK-2 cells. NLRP3 expression and caspase-1 and IL-1*β* cleavage were enhanced by exposure to glucose in a dose-dependent manner and peaked at a glucose concentration of 50 mM (Figures 4(a) and 4(b)). However, at this concentration, we observed morphological alterations in HK-2 cells. We therefore used a glucose concentration of 35 mM in subsequent experiments. HK-2 cells were treated with 35 mM glucose for 72 h, and NLRP3 expression and caspase-1 and IL-1*β* cleavage were found to change in a time-dependent manner, with peaks at 48 h (Figures 4(c) and 4(d)).

### 3.8. Effect of Curcumin on Fibronectin Expression and NLRP3 Inflammasome Activation in HK-2 Cells Exposed to High Glucose Concentrations

**Figure 5:**
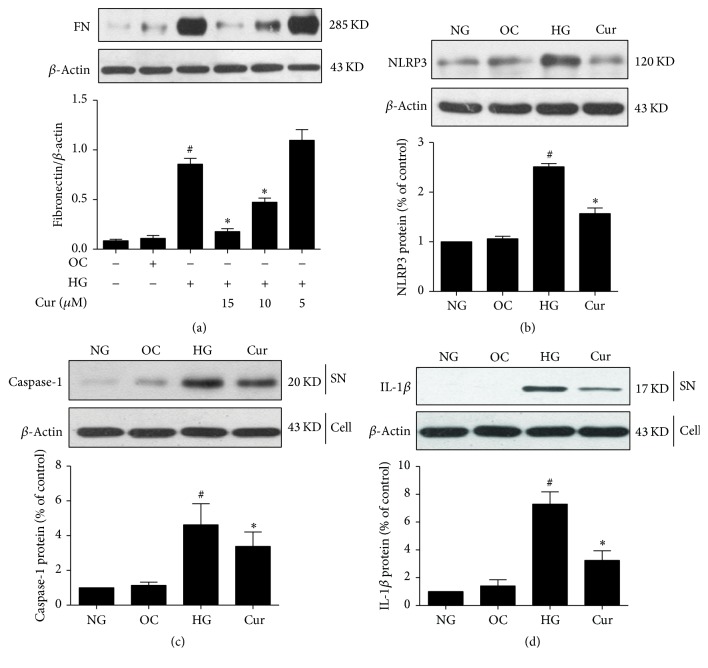
Curcumin inhibited the high glucose-induced increases in fibronectin and NLRP3 expression and NLRP3 inflammasome activation in HK-2 cells as assessed using Western blot analysis. HK-2 cells were assigned to four groups: (1) normal glucose (NG); (2) high glucose (HG); (3) curcumin (Cur); and (4) osmotic control with mannitol (OC). (a) Fibronectin protein expression in HK-2 cells exposed to 35 mM glucose for 48 h with or without different doses of curcumin. (b) NLRP3 protein expression in HK-2 cells treated with 35 mM glucose for 48 h and 10 *μ*M curcumin. (c, d) Cleaved caspase-1 p20 and 17 kD mature interleukin-1*β* (IL-1*β*) in culture supernatants. *β*-Actin was used as the internal loading control. ^#^*p* < 0.05, compared with the 5 mM glucose (NG) group; ^*∗*^*p* < 0.05, compared with the 35 mM (HG) group. The average values from three independent experiments are shown.

HK-2 cells stimulated with 35 mM glucose were treated with different curcumin doses for 48 h. The high fibronectin expression induced by glucose stimulation was significantly reduced after curcumin treatment at doses of 10 and 15 *μ*M; however, cell apoptosis was observed at the latter dose (Figure 5(a)). Western blot analysis showed that NLRP3 expression and caspase-1 and IL-1*β* cleavage were higher in HK-2 cells treated with 35 mM glucose than in cells treated with the same dose of mannitol. These changes were inhibited in cells treated with 10 *μ*M curcumin (Figures 5(b)–5(d)). Neither NLRP3 expression nor caspase-1 and IL-1*β* cleavage were affected by mannitol stimulation (Figure 5).

## 4. Discussion

The present study evaluated the effects of curcumin treatment on DN in db/db mice and investigated the underlying mechanisms in HK-2 cells. The results showed that curcumin treatment decreased renal hypertrophy, improved renal function (as assessed using the urinary albumin excretion rate), and ameliorated renal histological changes in db/db mice. In addition, the findings in HK-2 cells suggested that the renoprotective effects of curcumin were mediated by the inhibition of NLRP3 inflammasome activation.

Curcumin, the yellow pigment of the plant* Curcuma longa* (turmeric), is extensively used in food preparation and has attracted considerable research attention for its various bioactivities [22, 23]. Curcumin has been well established to exert a variety of pharmacological activities, in particular anti-inflammatory activity [13]. The renoprotective effect of curcumin in streptozotocin-induced DN rats has been confirmed [24, 25]. In these rats, curcumin alters posttranslational modifications of histone H3, heat-shock protein-27, and MAP kinase p38 expression [26] and prevents diabetes-associated abnormalities in the kidneys by inhibiting p300 and nuclear factor-*κ*B [27]. However, streptozotocin-induced DN rats are a model of type 1 diabetes, and little is known about the role of curcumin in db/db mice, which are a model of type 2 diabetic mellitus. The db/db mice are a genetic model of obesity associated with hyperphagia and hyperglycemia. A major benefit of this model is the consistent temporal development of hyperglycemia (6–8 weeks), albuminuria (10–12 weeks), and mesangial matrix expansion (14–16 weeks) [28]. Moreover, the glomerular mesangial expansion and histological lesions in these mice resemble those found in human DN.

One of the hallmarks of DN is the development of proteinuria, which is usually followed by a progressive decline in renal function. Urinary albumin excretion is regarded as one of the earliest signs of glomerular damage in overt diabetic renal disease. This damage is characterized structurally by glomerular basement membrane thickening and mesangial expansion, which can be detected histologically as an increase in the PAS-positive mesangial matrix area, due to the accumulation of extracellular matrix proteins. In this study, we found that elevated urinary albumin excretion, mesangial matrix expansion, and the upregulated expression of collagen IV and fibronectin were lower in the curcumin group than in the mock treatment group. In addition, remarkable changes were observed in body weight, kidney weight, and SCR levels after curcumin treatment. These findings indicate that curcumin inhibited disease progression in the early stage of DN in db/db mice. Furthermore, our study demonstrated that blood glucose levels were not affected by curcumin treatment, indicating that the renoprotective effects of curcumin are independent of any hypoglycemic action in this animal model of DN.

We speculated that the renoprotective effects of curcumin may be mediated by the suppression of NLRP3 inflammasome activation and IL-1*β* production. This is because curcumin has been shown to reduce NLRP3 inflammasome activation and IL-1*β* production in lipopolysaccharide-induced septic shock [29], and both IL-1*β* (a proinflammatory cytokine) and NLRP3 inflammasomes have been shown to be involved in the development of DN [14, 17]. Furthermore, recent reports have implicated that the NLRP3 inflammasome is an important contributor to inflammation and tissue damage during acute kidney injury, chronic kidney disease [30], and kidney inflammation and fibrosis [31]. Correa-Costa et al. [32] confirmed the important roles of both Toll-like receptors and NLRP3 inflammasomes in an experimental model of tubulointerstitial nephritis and found that allopurinol downregulated individual components of the inflammasome pathway and diminished this injury. Together, these results indicate that the NLRP3 inflammasome is a potential therapeutic target in DN.

Consistent with the aforementioned reports, our study showed that NLRP3 expression and caspase-1 and IL-1*β* cleavage were significantly inhibited in curcumin-treated db/db mice, suggesting that curcumin may attenuate DN progression by inhibiting NLRP3 inflammasome activation. To further evaluate this, we observed the effects of curcumin treatment on NLRP3 inflammasome activity in HK-2 cells exposed to high glucose levels. We selected HK-2 cells, which are immortalized proximal tubular epithelial cells, rather than mesangial cells because tubulointerstitial inflammation is crucial in promoting the development and progression of DN [33] and because NLRP3 protein is mainly expressed in renal tubular epithelial cells [14]. We found that NLRP3 expression and caspase-1 and IL-1*β* cleavage were inhibited after curcumin treatment in HK-2 cells exposed to high glucose levels, showing that curcumin inhibits the activation of NLRP3 inflammasomes.

Our study has some limitations. Curcumin is poorly absorbed when administered orally and has low solubility in water. Moreover, we did not assess the pharmacokinetics of curcumin or measure the plasma and tissue concentrations of curcumin in the mice. Nevertheless, we did obtain measurable changes in the parameters tested after curcumin treatment. It would be interesting to change the curcumin dosage or route of administration in further experiments in order to improve the bioavailability of curcumin and perhaps obtain better results.

In conclusion, curcumin displays renoprotective effects after disease onset in db/db mice despite sustained hyperglycemia. The protective effect of curcumin in DN may be attributable, at least in part, to the inhibition of NLRP3 inflammasome activation. Collectively, our findings may open a new avenue to explore the effects and molecular mechanisms of curcumin in DN.

## Figures and Tables

**Table 1:** Curcumin protects against the progression of diabetic nephropathy in db/db mice.

	db/m	db/db	db/db + Cur
Body weight, g	20.11 ± 1.38	44.91 ± 5.57^#^	35.54 ± 3.02^*∗*^
Kidney weight, g	0.14 ± 0.02	0.23 ± 0.04^#^	0.19 ± 0.03^*∗*^
Blood glucose, mmol/L	7.86 ± 0.98	27.44 ± 2.97^#^	26.34 ± 5.74
SCR, *μ*mol/L	38.48 ± 7.39	49.48 ± 5.59^#^	41.90 ± 4.71^*∗*^
BUN, mmol/L	8.89 ± 2.10	9.58 ± 2.13	9.34 ± 2.30
Urinary albumin, *μ*g/24 h	38.08 ± 23.37	603.83 ± 234.22^#^	243.14 ± 50.27^*∗*^

db/m, nondiabetic mice; db/db, diabetic mice (mock-treated); db/db + Cur, db/db mice treated with curcumin at a dose of 200 mg/kg body weight.

^#^ *p* < 0.05, compared with the db/m mice (control); ^*∗*^*p* < 0.05, compared with the mock-treated db/db mice (*n* = 6 per group).

Values shown are mean ± SD.

SCR, serum creatinine; BUN, blood urea nitrogen.
